# GH Responsiveness in Children With Noonan Syndrome Compared to Turner Syndrome

**DOI:** 10.3389/fendo.2021.737893

**Published:** 2021-11-09

**Authors:** Jovanna Dahlgren, Kerstin Albertsson-Wikland

**Affiliations:** ^1^ Gothenburg Paediatric Growth Research Centre (GP-GRC), The Institute of Clinical Sciences, The Sahlgrenska Academy at the University of Gothenburg, Gothenburg, Sweden; ^2^ Department of Physiology/Endocrinology, The Institute of Neurosciences and Physiology, The Sahlgrenska Academy at the University of Gothenburg, Gothenburg, Sweden

**Keywords:** growth hormone, growth response, height, IGFI, IGFBP3, Noonan syndrome, Turner syndrome, PTPN11

## Abstract

**Background:**

Despite different genetic background, Noonan syndrome (NS) shares similar phenotype features to Turner syndrome (TS) such as short stature, webbed neck and congenital heart defects. TS is an entity with decreased growth hormone (GH) responsiveness. Whether this is found in NS is debated.

**Methods:**

Data were retrieved from combined intervention studies including 25 children diagnosed with NS, 40 diagnosed with TS, and 45 control children (all prepubertal). NS-children and TS-girls were rhGH treated after investigation of the GH/IGFI-axis. GH was measured with poly- and monoclonal antibodies; 24hGH-profile pattern analysed by PULSAR. The NS-children were randomly assigned to Norditropin^®^ 33 or 66 μg/kg/day, and TS-girls were consecutively treated with Genotropin^®^ 33 or 66 μg/kg/day.

**Results:**

Higher PULSAR-estimates of 24h-profiles were found in both NS-children and TS-girls compared to controls: Polyclonal GH_max_24h-profile (Mean ± SD) was higher in both groups (44 ± 23mU/L, p<0.01 in NS; 51 ± 47, p<0.001 in TS; compared to 30 ± 23 mU/L in controls) as was GH-baseline (1.4 ± 0.6 mU/L in NS; 2.4 ± 2.4 mU/L in TS, p<0.01 for both, compared to 1.1 ± 1.2 mU/L in controls). Pre-treatment IGFI_SDS_ was 2.2 lower in NS-children (-1.7 ± 1.3) compared to TS-girls (0.6 ± 1.8, p<0.0001). GH_max_, IGFI/IGFBP3-ratio_SDS_, and chronological age at start of GH accounted for 59% of the variance in first-year growth response in NS.

**Conclusion:**

Both prepubertal NS-children and TS-girls had a high GH secretion, but low IGFI/IGFBP3 levels only in NS-children. Both groups presented a broad individual response. NS-children showed higher response in IGFI and growth, pointing to higher responsiveness to GH treatment than TS-girls.

## Introduction

Individuals with syndromes, like Turner syndrome (TS), have been reported to have reduced growth hormone (GH) responsiveness - partially overcome by higher rhGH dosage (1). Moreover, the growth response in short children receiving recombinant human (rh)GH is highly variable independent on diagnosis but dependent on the individual GH responsiveness, which can be calculated based on the growth response as well as IGFI response on rhGH (2, 3).

Due to the phenotypical resemblance, NS has previously been called “male Turner syndrome (TS)” (4), despite different genetic background. Both syndromes are characterised by short stature, pterygium colli, webbed neck, cardiac anomalies, pectus deformities, and mild hearing loss. Usually, NS males suffer from cryptorchidism, while TS girls most often fail to enter puberty and are infertile (5, 6).

One of the most predominant features of children with NS and TS is short stature affecting more than 80% (7). Possible answers to the cause of poor growth in patients with NS include disturbances in GH/insulin-like growth factor-I (GH/IGFI) axis. Findings are however contradictory and both low (8, 9), normal (10) or high (11, 12) spontaneous GH secretion has been reported, as well as either low (13–16) or normal (17, 18) GH_max_ levels in response to stimulatory test. Still, all of these studies found lower level of IGFI.

So far, only two studies have displayed detailed analyses pattern of the spontaneous GH secretion and its correlation with growth in NS patients treated with rhGH. Both Ahmed et al. (11) with a pilot study of four patients, and Noordam et al. (8) with 16 prepubertal NS patients, showed a disorganised pattern categorized by irregular wide pulses and high trough concentrations in overnight GH secretion profiles particularly in boys with NS. However, despite the evidence for disturbed GH/IGFI axis, the patients responded to rhGH treatment, showing an increase in both height velocity and in IGFI level during one year of treatment.

Our overall aim was to investigate GH/IGF status, response in growth and IGFI, and responsiveness to rhGH treatment in children with NS compared to TS. In this paper, we present unique and detailed data on the 24hGH profile in 25 prepubertal children with NS, and we compare these data to that of girls with TS and to healthy normally growing children as a control group. Both growth response and response for IGFI and IGFBP3 during the first year of GH treatment were measured and compared. Our hypothesis was that children with NS have disturbed GH secretion but normal GH responsiveness, giving greater response on a similar dose of rhGH treatment compared to girls with TS.

## Materials and Methods

### Study Subjects

*Noonan children:* This study group comprised 25 prepubertal patients with NS (13 girls, 12 boys) all included in the formal trial GHN00-1658 and were followed prospectively from pre-treatment to adult height. The entry criteria were postnatal growth restriction leading to short stature, webbed neck, ptosis, facial dysmorphism, low posterior hairline, congenital heart defects but no cardiomyopathy, normal karyotype, hematologic anomalies, normal liver and kidney function and delayed bone age (19). All NS were evaluated for the PTPN11 mutation at the genetic lab of the Sahlgrenska hospital, Gothenburg, Sweden. Sixteen NS children had mutations in the PTPN11 gene. If negative result, also BRAF, CBL, HRAS, KRAS, NRAS, RAF1, MAP2K1, MAP2K2, SHOC2, SOS1, NF1 and SPRED1 genes were analysed with HaloPlex target enrichment (Agilent) and then with next generation sequencing (MiSeq, Illumina) by Professor Marie-Louise Bondeson at the genetic lab of Uppsala University, Uppsala, Sweden. Five NS children had common mutations in NS (two in SOS1, one in BRAF, one in the SHOC2 gene, and one in NRAS) but in four no genetic mutation was found. All 25 patients remained prepubertal for at least one year during treatment.

*Turner girls*: The TS group included 40 prepubertal girls without other diseases and not treated with other hormones than rhGH, followed prospectively from pre-treatment year to one year of treatment. They were consecutively enrolled at GP-GRC within four multicentre studies (TRN 88-072; TRN 87-052) (20–22). All TS were genetically evaluated and 31 found to have a karyotype with compete absence of one X-chromosome (XO), five with either isochromosome or ring chromosome, one with 45x deletion and in the remaining four mosaicism, partial in X, (XX/XO).

*Healthy children:* Data from a reference group of 45 healthy children (12 girls, 33 boys) with normal height (**Table 1**) were also used (24).

**Table 1:** Auxological data and 24hour GH profile measured with polyclonal antibodies in 25 with Noonan syndrome (NS) (with PTPN11 mutation (n=16, without n=9) and in 40 with Turner syndrome (TS) (XO in 31 and 9 either mosaicism or isochromosome), compared to children with normal height (n=45).

Patients	Age (years)	Height (SDS)^a^	Baseline GH (mU/L)	GH mean (mU/L)	GH max (mU/L)	Peaks(n/24h)	Peak height (mU/L)	AUC_b_(mU/L 24h)	AUCt(mU/L 24h)
**NS Total** **NSPTPN11** **NS other genes**	8.3±2.6**(3.1-13.8) 8.6±1.8** (4.9-13.8) 7.1±2.2 (3.1-12.7)	-2.7±0.8*** (-4.0-(-0.7)) -3.0±0.5 (-4.0-(-2.4)) -2.4±0.7 (-3.3-(-0.7))	1.4±0.6** (0.7-2.7) 1.5±0.6 (0.7-2.7) 1.3±0.65 (0.7-2.4)	6.3±2.0** (3.1-10.8) 6.1±1.4 (3.1-10.8) 6.1±2.2* (3.9-10.0)	44±23* (14-104) 43±11 (14-103) 41±26 (19-104)	9.7±2.1* (6-14) 9.7±1.6* (7-12) 9.9±2.6 (6-14)	14.8±5.5 (6.6-26.8) 13.9±3.4 (6.4-25) 14.8±6.5* (7.5-27)	117±47* (38.1-227) 111±33(38-227) 113±50 (63-194)	151±48** (74.3-253) 146±33* (74-253) 145±52 (85-233)
**TS Total** **TS 45X0** **TS mosaic/isochromos**	7.2±2.8 (3.1-13.4) 6.8±2.9 (3.7-13.4) 8.4±2.7 (3.1-11.0)	-2.8±0.7 (-4.4-(-1.2)) -2.7±0.9 (-4.4-(-1.2)) -3.0±0.45 (-3.9-(-2.3))	2.4±3.1 (0.1-17.5) 2.4±3.1 (0.2-17.5) 2.3±3.6 (0.1-11.1)	7.8±4.0 (2.4-20) 8.2±3.8 (3.0-20) 6.4±4.4 (2.4-16)	51±47 (14.2-302) 56±52 (16-302) 35±12 (14-54)	8.6±2.0 (5-12) 8.4±2.0 (6-12) 9.0±2.1 (5-12)	19.0±9.6 (5.1-56.2) 20±9.9 (5.1-56) 14.9±7.7 (6.3-29)	131±66.3 (51.3-389) 140±71* (58-389) 100±35 (51-144)	187±95.4*** (58.7-480) 197±92** (72-480) 154±104 (59-397)
**Healthy controls**	10.6±1.8 (5.8-13.5)	0.0±0.9 (-1.5-1.4)	1.1±1.2 (0.1-6.7)	5.0±3.0 (1.4-15.5)	30.1±23.3 (7.4-140)	8.0±2.5 (4-15)	14.1±8.0 (4.5-39.0)	94±56.7 (26.5-306)	120±73.0 (33.1-379)

*p < 0.05; **p < 0.01, ***p < 0.001 compared to the healthy control group; a) Swedish references (23).

Results are expressed as mean±SD (range).

Auxological data and 24hGH profile are presented subdivided based on genotypes in **Table 1**. Height for prepubertal children was calculated from the prepubertal childhood component, which gives an accurate estimate of height as it does not underestimate height if delayed puberty (23) of the Swedish reference (25).

All children were investigated at GP-GRC at Queen Silvia Children’s Hospital, Gothenburg, Sweden. The growth of all patient groups has been followed since birth at Well Baby clinics and in schools in Sweden. Puberty was accessed according to Tanner for breast and PH and Prader for testicular volume. Data were only included for prepubertal individuals, ie. breast stage 1 or testicular volume <4ml, was used.

### Study Protocol

All participants were admitted to the hospital the day before carrying out the 24hGH profile. During the two days of hospitalisation they were encouraged to have normal activity, sleep and received a normal diet. A constant withdrawal pump (Swemed, Göteborg, Sweden) with non-thrombogenic catheter (Carmeda AB, Stockholm, Sweden) were used to collect blood samples as previously described (24). The heparinized tubes were changed every 20 min for 24h, giving a total of 72 integrated 20min samples in all healthy children, the TS girls, and in 8 NS children. In the remaining 17 NS children, tubes were changed every 30minute giving 48 samples per individual, previously evaluated to equally capture the 24hGH pattern (26).

The IGFI and IGFBP3 serum concentrations were measured before starting treatment (n=25 for NS and n=30 for TS), at an average of 10 days (range between 7-21 days) in 15 NS and in 24 TS, and also after one year of treatment in 23 NS and in 24 TS girls. These blood samples were collected after noon, and approximately 24h after the latest GH injection.

Biosynthetic rhGH (Norditropin^®^/Novo Nordisk in all NS and Somatonorm^®^ in 4 or Genotropin^®^/Pfizer in 36 TS) was given in a dose of 33 µg/kg/day in 9 patients with NS (5 girls, 4 boys) and in 32 patients with TS, and 66 µg/kg/day in 16 patients with NS (8 girls, 8 boys), and in 8 patients with TS. The dose was randomized in 16 NS, whereas the dose in TS was chosen consecutively with a lower dosage of 33 µg/kg/day in the first 32 patients and later with a higher dosage of 66 µg/kg/day in 8 patients. The doses were adjusted according to changes in body weight every 3 months. Details on NS group are reported elsewhere (19).

### Measurements

#### GH Measurements

Endogenous serum GH concentrations were measured using a polyclonal antibody-based immuno-radiometric assay using the WHO First International Reference Preparation (IRP) 80/505 (Pharmacia Diagnostics AB, Uppsala, Sweden). During 1991 and 1992 the laboratories in Sweden switched from WHO IRP 66/217 to WHO IRP 80/505; a conversion factor of 1.55 had to be used to compare the results on GH levels (27). All girls with TS had their GH measurements performed both with polyclonal (IRMA) and monoclonal (trIFMA) immuno-radiometric assay, and ten profiles from children with NS were also analyzed with both methods (8 with 72 20-min and 2 with 48 30-min integrated samples). For all other comparisons presented in tables, figures and result section, the results from the polyclonal IRMA were used.

The analysis of the 24hGH profiles was performed as previously reported for pulse detection and peak analysis with the PULSAR program, with settings for GH-profiles (22, 28). PULSAR gave for the 24hGH profile mean, max, the area under the plasma GH concentration curve over zero level, (AUCt), the AUC over GH-baseline level (AUCb), GH-baseline levels (the level in between the peaks), the number of GH peaks, the peak height (from zero line) and peak width. We present data in tables and figures with mU/L, using the unit-references WHO IRP 66/217 and WHO IRP 80/505, which should be divided by 3 for results with microgram/L.

#### *IGFI* and *IGFBP3 Measurements*

The serum concentrations of IGFI were measured at start of GH treatment, at an average of 10 days (range 7-21 days), and at 12 months after start of GH treatment. An IGF-binding protein (IGFBP)–blocked radioimmunoassay (RIA) without extraction and in the presence of an approximately 250-fold excess of IGFII (Mediagnost GmbH, Tübingen, Germany) was used (29). Serum IGFBP3 concentrations were determined at the same times as IGFI using previously reported RIA method (Mediagnost GmbH, Tübingen, Germany) (30). IGFI and IGFBP3 were converted in SDS using reference values for healthy children (31) as well as the ratio IGFI/IGFBP3 (31).

### Ethics

The Ethic Committee of the Faculty of Medicine at the University of Gothenburg approved the Noonan study GHN00-1658 (Dnr 208-05), the healthy children study (Dnr 129-85), and for the Turner studies TRN 88-072 and TRN 87-052 the Gothenburg part (Dnr 142-85; Dnr 76-88; Dnr 456-94) of the multicentre studies approved by the Ethics Committee of Lund University. Informed consent was obtained from the parents and from patients if they were old enough.

### Statistical Analyses

All data are presented as mean ± SD, if not otherwise stated. Mann-Whitney U-test for continuous variables was used for comparison between two groups. Analyses were adjusted for pretreatment status and rhGH dose in the multiple regression analyses with a normally transformed dependent variable, Blom’s method. Spearman correlation coefficients and corresponding p-values were obtained when describing relation between two continuous variables.

To test the difference of slopes, for prediction of delta height_SDS_ with SDS for IGFI, IGFBP3 and the ratio, multiple regression analyses were done with interaction term of predictor and group variable (NS or TS) added to the model. These analyses were also adjusted for rhGH dose and the p-value for the interaction term tested if the slopes were significantly different between the two groups.

Finally, multiple stepwise regression models were performed for prediction of delta height_SDS_ first year of GH-treatment within NS and TS by initially including all significant predictors from univariate analyses. All tests were two-tailed and conducted at 5% significance level.

## Results

### A. Pretreatment Findings in GH/IGFI Axis

*GH profile*: The GH mean, GHmax, AUCt, AUCb, and the GH-baseline were significantly higher in both NS and TS compared to healthy children. The average number of GH peaks during a 24h period was higher only for NS patients compared with TS and healthy children, respectively. In the TS group, the peak width was statistically greater than in the healthy group. See **Table 1** for details of total group and split up into subgroups based on genetic background. The NS children with PTPN11 gene mutation had all Pulsar-estimates with exception of number of peaks, with greater values, when compared to the NS without PTPN11 gene mutation. The NS children with PTPN11 were shorter compared to those without this gene mutation (p<0.001).

**Figure 1:**
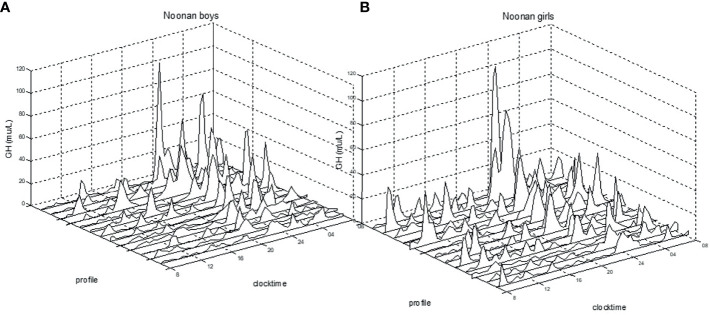
Individual 24h-GH profiles for prepubertal boys **(A)** and for girls **(B)** with Noonan syndrome. Clock time for integrated 20/30 min samples *versus* GH mU/L (divide by 3 for ug/L), measured with polyclonal antibodies.

When compared to healthy boys, boys with NS displayed significantly greater mean GH (6.2±1.8 *vs.* 4.6±2.3 mU/L; p<0.05), GHmax (46±23.9 *vs.* 29±25.5 mU/L; p<0.01), GH-baseline (1.5±0.7 *vs.* 1.0±1.2 mU/L; p<0.05), number of peaks (9.5±1.9 *vs.* 7.6±2.1; p<0.05), AUCb (114.3±41.4 *vs.* 85.0±45.5 mU/L; p<0.05), and AUCt (150.1±42.1 *vs.* 110.1±56.5 mU/L; p<0.05). Girls with NS did not show any significant difference in the 24hGH profile when compared to healthy girls. See **Figure 1** for the large variation in individual patterns of the 24hGH profiles in NS girls and NS boys, respectively.

**Figure 2:**
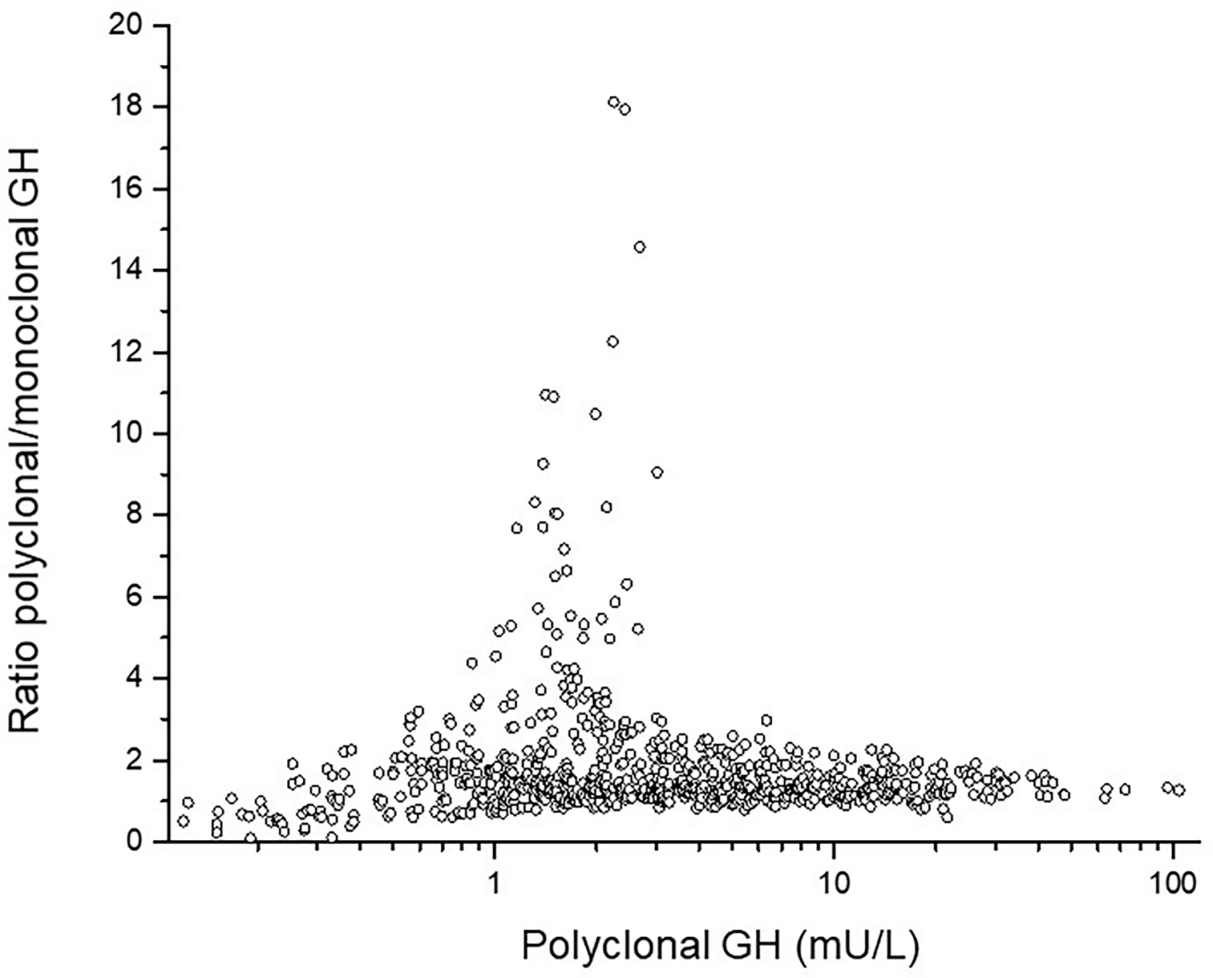
The individual ratio polyclonal/monoclonal isoforms of GH in the y-axis *versus* total polyclonal GH antibodies in the x-axis. Notice the high ratio between 1 and 3 mU/L corresponding to 0.3 and 1 ug/L.

*GH isoforms:* In ten out of the 25 children with NS, GH was measured both with polyclonal and with monoclonal assay (the latter detecting only the 22kDa-form). The polyclonal/monoclonal GH ratio had extremely high levels of non-22kDa-isoforms in three females and one male. For the entire NS-group, this ratio was for GHmax 1.13 ± 0.7, for 24hGH mean 1.38 ± 1.1, for AUCt 1.38 ± 1.1 and for GH-baseline 1.85 ± 1.3. The GH concentration for all samples in the 24hGH-profile *versus* amount of non-22kDa GH are shown as polyclonal *vs.* the ratio polyclonal/monoclonal GH antibodies, see **Figure 2**. Notice the highest proportion of non-22-kDa GH isoforms in the range 1-3 mU/L, corresponding to 0.3-1 ug/L.

*IGFs:* Mean IGFI_SDS_, IGFBP3_SDS,_ and the IGFI/IGFBP3 ratio_SDS_ at start were statistically lower in NS than in TS (see **Table 2**). When splitting up into NS subgroups, the IGFI and IGFBP3 levels did not reach significant difference between the PTPN11 group and the non-PTPN11group, but both did, when comparing total NS *versus* total TS groups or comparing PTPN11 *versus* TS45x0 the NS had significant lower IGFI levels before starting rhGH treatment.

**Table 2:** Comparison of changes in height, IGF1, IGFBP3, and IGF1/IGFBP3 ratio between Noonan (NS) and Turner groups (TS) on rhGH treatment.

	Noonan Group(n=25)	NS non-PTPN11 (n=9)	NS PTPN11 (n=16)	Adjusted p-value TPN11+ *versus* PTPN11-	Turner Group (n=40)	Turner 45X0(n=31)	Adjusted p-value NS total *vs* TS total	Adjusted p-valuePTPN11+ *versus* TS 45X0
**Height SDS** At start Δ 0-1 year **IGFI SDS** At start	-3.0±0.5 0.7±0.3 -1.8±1.1	-2.4±0.7 0.8±0.5 -2.2±1.5	-3.1±0.5 0.7±0.3 -1.6±1.1	<.01 0.68 0.45	-2.8±0.7 0.6±0.2 0.57±1.76	-2.7±0.8 0.5±0.2 0.63±1.83	0.16 0.023 <.0001	0.15 0.023 <-0001
Δ 0-10 days	1.96±1.1	2.45±1.0	1.45±1.1	0.40	0.98±2.04	0.79±1.90	0.44	0.46
Δ 0-1 year	3.17±1.2	4.04±1.2	2.50±1.3	0.01	1.90±1.87	1.68±1.88	0.03	0.14
**IGFBP3 SDS** At start	-1.23±1.0	-1.50±2.0	-1.30±1.1	0.49	0.70±1.99	0.80±1.99	<.0001	<.0001
Δ 0-10 days	1.85±1.4	2.34±1.0	1.46±1.4	0.17	0.95±1.81	0.91±1.85	0.60	0.35
Δ 0-1 year	2.05±0.8	2.93±1.0	1.81±0.7	0.08	2.00±2.34	1.91±2.40	0.006	0.36
**IGFI/IGFBP3 SDS** At start	-1.23±1.0	-1.50±0.9	-0.87±0.9	0.14	0.19±1.33	0.18±1.46	<0.001	0.014
Δ 0-10 days	0.91±0.78	1.11±0.6	0.42±0.9	0.40	0.43±1.89	0.24±1.74	0.19	0.90
Δ 0-1 year	2.14±0.97	2.77±1.0	1.54±1.29	0.04	0.69±1.84	0.50±1.82	0.43	0.06

Δ = delta.

Comparisons done with Mann-Whitney U-test.

Results are express as mean±SD. P-value was adjusted with Blom’s method for differences in IGFI SDS at start.

### B. Growth and IGFI Response to GH Treatment in NS and TS

Changes in SDS from before GH start to one year in IGFI, IGFBP3, and IGFI/IGFBP3-ratio were greater in NS than TS, See **Table 2** for more details. The differences between groups were still statistically significant after adjustment for differences in pre-treatment values and rhGH dose, but lost significance for the change in IGFI/IGFBP3-ratio_SDS_. When the NS PTPN11 group was compared with the TS 45X0 group the significant difference in changes over time between the total NS and TS groups was lost.

**Figure 3:**
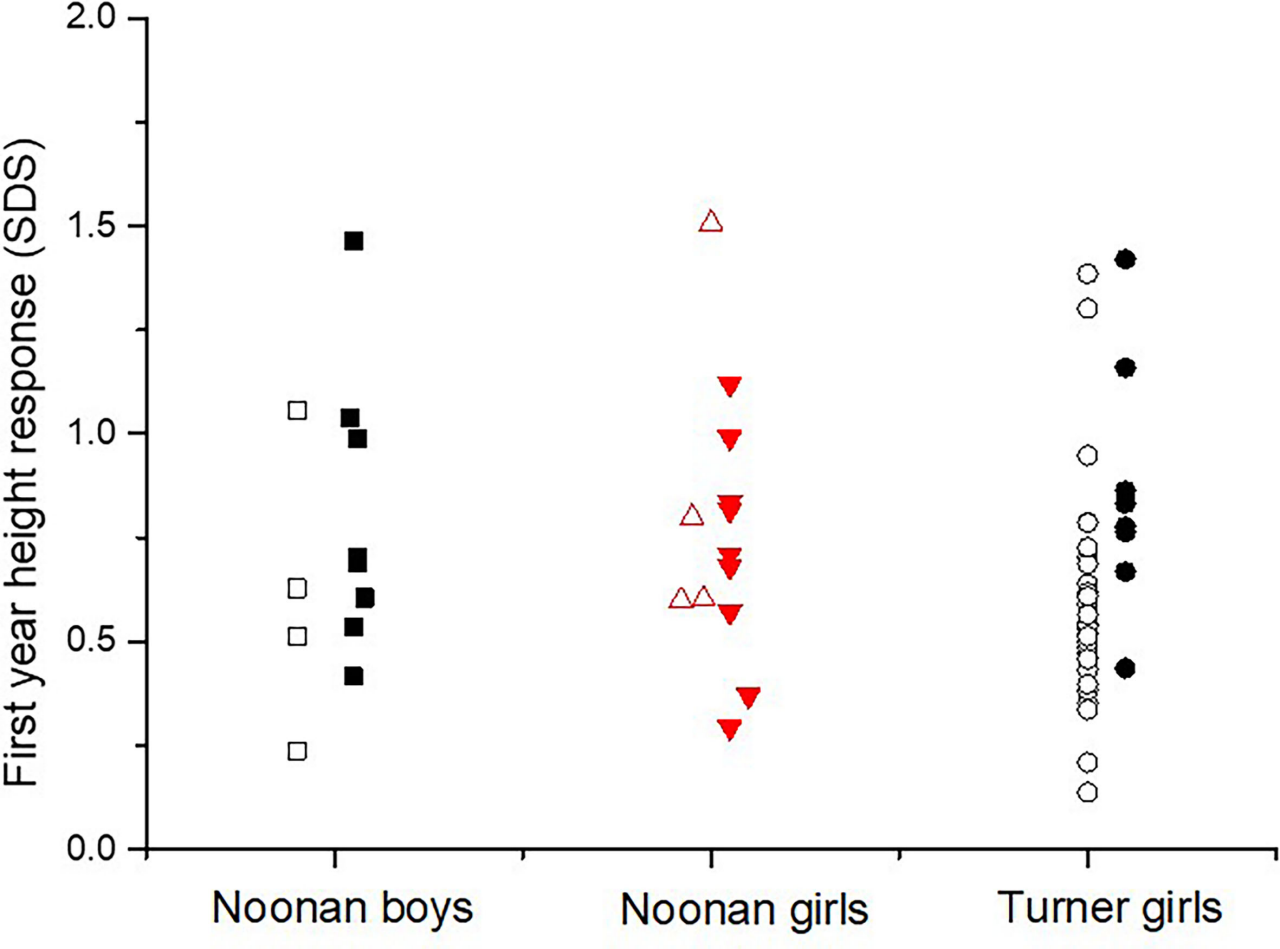
The individual growth response as the first-year delta height_SDS_ in diagnosed with Noonan or Turner syndrome subdivided by gender, on either rhGH 33 or 66 µg/kg/day (open figures respectively filled figures).

There was a significant higher growth response during the first year on rhGH comparing the totals NS group and TS group (p<0.023), see **Table 2**. Mean change in height during the first year on rhGH for NS boys was 0.74 ± 0.34 SDS and for NS-girls was 0.76 ± 0.32 SDS, compared to 0.69 ± 0.29 SDS for TS-girls (p=0.058). **Figure 3** shows the broad variation in individual first-year growth response to the two rhGH doses in NS and TS groups expressed as delta height_SDS_.

**Figure 4:**
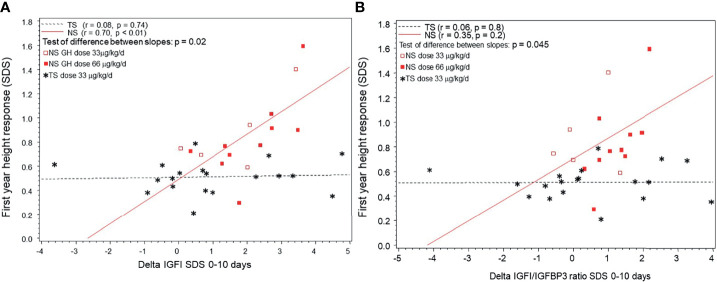
**(A)** The individual growth response as the first-year delta height_SDS_ correlated to short-term delta IGFI_SDS_ 0-10 days of rhGH in children diagnosed with Noonan syndrome (NS) or Turner syndrome (TS). NS are depicted with circles and TS with stars (in TS shown only for standard dose). **(B)** The individual growth response as the first-year delta height_SDS_ correlated to delta IGF1/IGFBP3 ratio_SDS_ 0-10 days of rhGH in children diagnosed with Noonan syndrome (NS) or Turner syndrome (TS). NS are depicted with circles and TS with stars (in TS shown only for standard dose).

In NS but not in TS patients first-year changes in height_SDS_ correlated positively with short-term changes in IGFI_SDS_ and IGFI/IGFBP3-ratio_SDS_ (see **Figures 4A, B**). When slopes were compared, first-year growth response in NS but not in TS correlated with the delta IGFI_SDS_ and delta IGFI/IGFBP3-ratio_SDS_ 0-10 days.

*For univariate correlations* between pre-treatment variables and growth response, see **Table 3**. In children with NS, growth response during the first year of rhGH treatment correlated positively with pre-treatment GH-baseline levels and IGFI_SDS_ at start and negatively with age at start. In TS, however, and contrary to NS, growth during the first year of rhGH treatment correlated negatively with pre-treatment GH-baseline levels, GH mean, peak height and AUCt.

**Table 3:** Correlation coefficients for the first-year growth response as dependent variable, using age, different GH and IGF variables at start or at 10 days of treatment in Noonan group and Turner group, respectively.

	Noonan group		Turner group	
Predictors	r*	p	r*	p
Age at start of GH	-0.53	0.006	-0.15	ns
**24hour GH profile values before start**				
GH baseline	0.63	0.0007	-0.33	0.04
GH mean	0.01	ns	-0.41	0.009
GHmax	-0.14	ns	-0.21	ns
number of peaks	0.14	ns	-0.01	ns
peak height	-0.19	ns	-0.33	0.04
AUCt	0.02	ns	-0.42	0.007
**At start**				
IGFI SDS	-0.49	0.01	-0.28	ns
IGFBP3 SDS	-0.07	ns	-0.35	0.06
IGFI/IGFBP3 SDS	-0.47	0.02	-0.19	ns
**At 10 days**				
Δ IGFI SDS	0.70	0.004	0.08	ns
Δ IGFBP3 SDS	0.59	0.02	0.06	ns
Δ IGFI/IGFBP3 SDS	0.35	ns	0.06	ns

*by Spearman correlation coefficient. ns, non-significant.

*In the multivariable regression* model for the NS group, GH-baseline level (p=0.001), IGFI/IGFBP3 ratio_SDS_ at start (p=0.033), and chronological age at start of treatment (p=0.037), accounted for 59% of the variance in the first-year growth response. For the TS group, the rhGH dose (p = 0.0091) and the mean 24hGH (p = 0.022) explained 28% of the variance.

## Discussion

The main finding of the present study was that NS-children showed higher response in IGFI as well as in growth, pointing to higher responsiveness to GH treatment compared to TS-girls.

In the NS-children but not in the TS-children these two GH effects showed great correlation.

The magnitude of mean growth response, independent of diagnosis and GH dose, was in parallel with findings in other patients with short stature without GH deficiency (32–34). Girls diagnosed with TS did not show as great response for IGFI as found in NS, whereas for growth the response was almost in parallel with similar doses of rhGH treatment, confirming findings by Romano et al. (16).

In our study, 60% of the variance in the first-year prepubertal growth response in NS could be explained by the chronological age at start, the GH-baseline level and the IGFI/IGFBP-3 ratio_SDS -_the three selected pre-treatment variables by the multivariable regression models. In girls with TS, the rhGH dose and mean 24hGH became the selected variables explaining only 28% of the variance. The influence of rhGH dose in TS is in accordance with the KIGS outcome research database (1, 35).

NS boys showed several differences in the pattern of the 24hGH profile with higher peaks and trophs resulting in an overall higher 24hGH concentration when compared to healthy boys, whereas girls in the NS group had similar GH concentrations to normal girls. Despite the physiological positive feedback of the low IGF serum concentrations stimulating endogenous GH secretion, rhGH treatment resulted in normalized IGF concentration and prepubertal growth in both boys and girls with NS.

The approach of comparing NS to TS revealed that after adjustment for rhGH dose and GH-status, the strong correlation between changes in height_SDS_ with the changes in IGFs already after 10 days of rhGH treatment was significant in NS only. Indeed, the examination of short-term changes during the first weeks could be regarded as an IGFI generation test (36, 37). As shown in both non-GH deficient and GH deficient children the short-term changes in IGF, IGFBP3, and their ratio, were more informative for short and long-term growth response than the obtained IGFI_SDS_-level per se on rhGH treatment (38, 39).

The lower increment in IGFI_SDS_ and IGFI/IGFBP3-ratio_SDS_ in TS could be interpreted as a substantial insensitivity for the IGF-response to GH in TS. This possibility was further supported by the fact that girls with TS, showed higher mean GH level, GH-baseline levels, amplitude, and number of GH peaks when measured with polyclonal antibodies (40). The abnormal proportion of non-22kDa GH isoforms (less active for growth) in TS than in normal growing children (41, 42) could be another explanation to a lower activation of the GH receptor in TS. Interestingly, here we found in four out of ten children with NS abnormally high proportion of non-22kDa isoform.

In both NS and TS, we found signs of an elevated GH secretion as measured with polyclonal antibodies, whereas the pulsatile pattern of GH with higher number of peaks was abnormal only in NS. These results are in accordance with findings by Noordam et al. (8) in 17 prepubertal NS children (13 boys and four girls) who also showed disturbances in the pattern of overnight GH secretion. They found low spontaneous GH max and high GH-baseline levels measured with overnight GH profiles using PULSAR (8), see review by Albertsson-Wikland and Rosberg (43). Half of their children had a mean overnight GH concentration below the lower limit of the normal range, whereas six instead had high GH baseline. Abnormalities in the GH secretion pattern with higher numbers of peaks and elevated GH-baseline could be due to a disturbance at the hypothalamic level such as an impaired balance between GH releasing hormone and somatostatin activity as implied from the present findings in Noonan boys. The fact that the mean GH level and the GH-baseline level are high could also indicate a partial resistance at GH post-receptor level and a reduced negative feedback mechanism.

A broad variation in individual growth response, independent of given rhGH dose, was found for both groups with NS and TS. This was previously found in children with other diagnoses as isolated GH deficiency, idiopathic short stature and small for gestational age (32–34). This is mainly caused by the individual responsiveness to GH, which can be mathematically estimated by prediction models for prepubertal growth response in children with GH deficiency, idiopathic short stature, small for gestational age (44, 45), and TS (1). For children with NS there is no specific model developed, leaving the clinician to use first-year growth response. We have previously shown that the first-year growth response incorporates the pre-treatment measures and can serve as a marker of individual responsiveness (3). In addition, the injection technique also influences the GH uptake (46). Daily deep subcutaneous injection is known to obtain high GH peaks and low/undetectable levels before next injection. Such a pattern has been shown to give an optimal signal for growth whereas continuous GH levels are more effective for IGF response (47).

In approximately 75% of children with NS, clinically diagnosed by phenotype, different gene mutations are detected (5). Most commonly, the PTPN11 gene mutation is found. In our group of 25 children with NS, we found somewhat higher proportions of identified PTPN11 *versus* other gene mutations and somewhat lower proportion of not identified genetic mutations. We subdivided the NS children into two groups - those with and those without the PTPN11 gene mutation – to explore the impact of the genetic basis on the GH/IGF axis and the GH responsiveness. The children with PTPN11 were shorter before treatment, thereby confirming precious results by Binder (48). However, no baseline IGFs or GH profile estimate differed according to the genetic background. On rhGH treatment, the two groups showed similar response for growth, whereas for IGFs those with PTPN11 showed a lower increment. Thus, we have not detected any impact of the various genetic bases in NS children for GH responsiveness for growth.

The study is not without limitations, such as the combination of data from several trials with somewhat different study design, although similar inclusion criteria. The different proportions of poly- and monoclonal antibodies would preferably been investigated in a majority of children for a stronger power and probably more significant results on growth response. A strength of the study was the relative high numbers of NS patients, besides 40 TS girls, that carefully were evaluated with a 24hGH profile and not only overnight sampling. Our one-centre study presents data on 12 males and 13 females with NS, which nearly reach the global previously published number of 30 NS patients being compared to TS (49). In addition, only two other papers with a total of 15 males and 6 females with NS have published spontaneous GH profiles overnight (8, 11). Another strength was that all participants remained prepubertal throughout the study period. This allowed accurate growth response evaluation without influence by pubertal growth, especially since it was estimated in relation to a prepubertal growth reference and not to a traditional total height reference with a mixed prepubertal/pubertal population for this age period (49).

## Conclusion

This is the first study in NS to evaluate the 24hGH secretion, as the pattern, amount of total as well as the proportion of 22kDa and non-22kDa GH forms, the IGFI response and the growth response to rhGH. We found children with NS to have disturbances in the secretion pattern of GH, with better growth response to treatment with rhGH than girls with TS. In parallel, children with NS show a greater IGFI increase as a marker of high responsiveness. Despite the overall good growth response, the broad individual responsiveness to rhGH must be considered for a more tailored successful treatment. For this purpose, we suggest the first-year height gain to be used for personalized GH-dosing.

## Data Availability Statement

The datasets for this article are not publicly available because too few individuals could be a legal problem in identifying personal details. Requests to access the datasets should be directed to JD, jovanna.dahlgren@gu.se.

## Ethics Statement

The studies involving human participants were reviewed and approved by Ethical committee at University of Gothenburg. Written informed consent to participate in this study was provided by the participants’ legal guardian/next of kin.

## Author Contributions

Both authors designed the study, developed the statistical analysis plan, and wrote the paper. KA-W is PI for all the studies used. All authors contributed to the article and approved the submitted version.

## Funding

This work was supported by the Governmental grants under the ALF agreement (ALFGBG-812951, ALFGBG-719041) and an unrestricted grant from NovoNordisk in Denmark. The authors declare that this study received funding from NovoNordisk. The funder was not involved in the study design, collection, analysis, interpretation of data, the writing of this article or the decision to submit it for publication.

## Conflict of Interest

Free drugs were given from NovoNordisk for NS and Pharmacia/Pfizer for those TS girls who received the higher rhGHdose. None of the pharmaceutical companies influenced or had any impact on the data analyses nor the writing of this manuscript.

The authors declare that the research was conducted in the absence of any commercial or financial relationships that could be construed as a potential conflict of interest.

## Publisher’s Note

All claims expressed in this article are solely those of the authors and do not necessarily represent those of their affiliated organizations, or those of the publisher, the editors and the reviewers. Any product that may be evaluated in this article, or claim that may be made by its manufacturer, is not guaranteed or endorsed by the publisher.
